# Total recall in the SCAMP cohort: Validation of self-reported mobile phone use in the smartphone era

**DOI:** 10.1016/j.envres.2017.10.034

**Published:** 2018-02

**Authors:** Michael O. Mireku, William Mueller, Charlotte Fleming, Irene Chang, Iroise Dumontheil, Michael S.C. Thomas, Marloes Eeftens, Paul Elliott, Martin Röösli, Mireille B. Toledano

**Affiliations:** a MRC-PHE Centre for Environment and Health, Department of Epidemiology and Biostatistics, School of Public Health, Imperial College London, W2 1PG, UK; b National Institute for Health Research Health Protection Research Unit in Health Impact of Environmental Hazards at King's College London and Imperial College London in partnership with Public Health England, Imperial College London, W2 1PG, UK; c Institute of Occupational Medicine, Edinburgh EH14 4AP, UK; d Department of Psychological Sciences, Birkbeck, University of London, WC1E 7HX, UK; e Department of Epidemiology and Public Health, Swiss Tropical and Public Health Institute, 4051 Basel, Switzerland; f University of Basel, 4001 Basel, Switzerland

**Keywords:** Mobile phones, Adolescents, Exposure measurement error, SCAMP

## Abstract

Mobile phone use, predominantly smartphones, is almost ubiquitous amongst both adults and children. However adults and children have different usage patterns. A major challenge with research on mobile phone use is the reliability of self-reported phone activity for accurate exposure assessment. We investigated the agreement between self-reported mobile phone use data and objective mobile operator traffic data in a subset of adolescents aged 11–12 years participating in the Study of Cognition, Adolescents and Mobile Phones (SCAMP) cohort. We examined self-reported mobile phone use, including call frequency, cumulative call time duration and text messages sent among adolescents from SCAMP and matched these data with records provided by mobile network operators (n = 350). The extent of agreement between self-reported mobile phone use and mobile operator traffic data use was evaluated using Cohen's weighted Kappa (ĸ) statistics. Sensitivity and specificity of self-reported low (< 1 call/day, ≤ 5 min of call/day or ≤ 5 text messages sent/day) and high (≥ 11 calls/day, > 30 min of call/day or ≥ 11 text messages sent /day) use were estimated.

Agreement between self-reported mobile phone use and mobile operator traffic data was highest for the duration spent talking on mobile phones per day on weekdays (38.9%) and weekends (29.4%) compared to frequency of calls and number of text messages sent. Adolescents overestimated their mobile phone use during weekends compared to weekdays. Analysis of agreement showed little difference overall between the sexes and socio-economic groups. Weighted kappa between self-reported and mobile operator traffic data for call frequency during weekdays was κ = 0.12, 95% CI 0.06–0.18. Of the three modes of mobile phone use measured in the questionnaire, call frequency was the most sensitive for low mobile phone users on weekdays and weekends (77.1, 95% CI: 69.3—83.7 and 72.0, 95% CI: 65.0–78.4, respectively). Specificity was moderate to high for high users with the highest for call frequency during weekdays (98.4, 95% CI: 96.4–99.5).

Despite differential agreement between adolescents’ self-reported mobile phone use and mobile operator traffic data, our findings demonstrate that self-reported usage adequately distinguishes between high and low use. The greater use of mobile smartphones over Wi-Fi networks by adolescents, as opposed to mobile phone networks, means operator data are not the gold standard for exposure assessment in this age group. This has important implications for epidemiologic research on the health effects of mobile phone use in adolescents.

## Highlights

• We assessed the validity of adolescents’ self-reported mobile phone use.
• The study accounted for weekday and weekend use of mobile phones separately.
• Findings show that self-reported usage distinguishes between high and low use.
• We recommend a combination of self-reported use over Wi-Fi & operator traffic data.
• Combined data will improve accurate exposure estimation in epidemiological studies.

## 1 Introduction

Mobile phone use is almost ubiquitous among adults and children, with over 90% ownership in adults and upwards of 75% in 12 to 15-year-olds in the UK (Ofcom, 2015). While these rates are comparable to those recorded a decade ago, children, particularly adolescents, now carry smartphones with myriad functions facilitating different and higher function usage: accessing the internet and sending text/instant messages on mobile phones are surpassing traditional telephony activities (Fowler and Noyes, 2015). There are potential health concerns with this increased use; whilst there is limited evidence available for adolescents, it is believed that, due to the continuous maturation of their nervous system and likely greater lifetime exposure, they may be especially susceptible to potential harmful effects from mobile phones (IEGMP, 2000). The International Agency for Research on Cancer (IARC) has classified radiofrequency electromagnetic fields (RF-EMF) as “possibly carcinogenic to humans” (IARC, 2011). However, the dose of RF-EMF that a regular mobile phone user is exposed to is difficult to measure as it is dependent on the frequency band, the power output of the mobile phone, and the duration and frequency of use. Depending on these parameters, a recent Swiss study in adolescents estimated an average whole body dose of 28 mJ/kg and an average brain dose of 190 mJ/kg from own mobile phone calls (Roser et al., 2017)

A major challenge with research on mobile phone use is the reliability of self-reported telephone activity to measure exposures, also known as recall error. Though imperfect, self-reported mobile phone use data are valuable, since objective data are often not available (Vergnaud et al., 2016). Validation studies of adults have revealed wide variation in the concordance between reported usage and objective network data (Abeele et al., 2013, Samkange-Zeeb et al., 2004). Similar studies in children and adolescents have found systematic errors, including self-reported call frequency being under and overestimated, and call duration consistently overestimated (Inyang et al., 2009, Aydin et al., 2011a, Kiyohara et al., 2015). Depending on the predominant direction of error, exposure misclassification could reduce, exaggerate, or invert a true association of mobile phone use with a given outcome (Vrijheid et al., 2009).

The purpose of this paper is to examine self-reported mobile phone usage data in relation to objective mobile operator traffic data in a subset of the SCAMP cohort (operator subset). This study represents one of the first to validate the accuracy of self-reported mobile phone use in adolescents, considering weekday and weekend use separately, and investigating the role of socio-demographic characteristics in relation to recall accuracy.

## 2 Materials and methods

SCAMP is a prospective school-based cohort study aimed to investigate cognitive and behavioural outcomes associated with use of mobile phones and other wireless technologies that emit RF-EMF. The SCAMP cohort directly addresses the WHO 2010 research agenda for radiofrequency fields that ranked prospective cohorts of children and adolescents as ‘highest priority research need’ (van Deventer et al., 2011). The cohort includes Year 7 pupils 11–12-years-old) across London, UK, from 39 secondary schools. Baseline data collection commenced November 2014 and was completed in July 2016. Data were collected from all adolescents in Year 7 (first year of secondary school) at each school in the cohort, unless parents or adolescents chose to opt out.

### 2.1 Self-reported mobile phone use

The method of data collection in SCAMP was a computer-based assessment that adolescents completed at school. The assessment includes a questionnaire that enquired about their use of mobile phones (e.g., number of calls, Short Message Service (SMS) text and instant messages sent) and other devices (e.g. time spent on laptop computers, video game consoles), and incorporated a battery of cognitive tests, in addition to wellbeing and behaviour scales. Questions on device use provided categorical responses for weekday and weekend use. For example, to measure the frequency of phone calls, adolescents who reported using or having used a mobile phone were asked separately for weekdays and weekend days, “How often do you make or receive calls with your mobile phone?”, and were given the following eight options, “Never”, “A few times per month”, “A few times per week”, “Approximately once per day”, “2–5 times per day”, “6–10 times per day”, “11–20 times per day”, and “21 or more times per day”. Similar response categories were provided for text and instant messages, and seven categories were presented for the daily amount of time spent talking on the phone, ranging from 0 min to 3+ hours daily.

### 2.2 Socio-demographic data

Demographic information, including age, sex, ethnicity (combined into “White”, “Black”, “Asian”, “Mixed” and “Other”), and parent occupation as an indicator for socioeconomic status (SES), was captured in the SCAMP assessment. We used the Office for National Statistics classification of parental occupation into five SES levels,^1^ with each child allocated the highest SES of either parent (Rose and Pevalin, 2010). To ensure sufficient numbers in each SES category and to be able to compare with earlier research (Aydin et al., 2011b), SES levels two and three, and four and five were collapsed into two SES classes,^2^ thereby creating high, medium, low SES categories.

### 2.3 Objective mobile operator traffic data on mobile phone use

Parents of children in the SCAMP cohort may provide consent to access health and education records, as well as objective mobile traffic data from network operators. Personal details of adolescents for whom we had parental consent were sent to the network operators for data linkage. Network operators matched the personal information to mobile operator traffic data by either using (1) the adolescent's mobile phone number plus either of the adolescent or the account holder's surname, date of birth or postcode; or (2) by using the adolescent's surname, date of birth and postcode should the phone number provided by the parent be incorrect. In this study, mobile operator traffic data were presumed to be the “gold standard” for mobile phone use in line with other validation studies (e.g., Shum et al., 2011; Heinävaara et al., 2011; Abeele et al., 2013). Of the four major network operators in the UK (Ofcom, 2016), three were contacted to obtain records of mobile phone use for those adolescents whose parents had given consent.

**Fig. 1:**
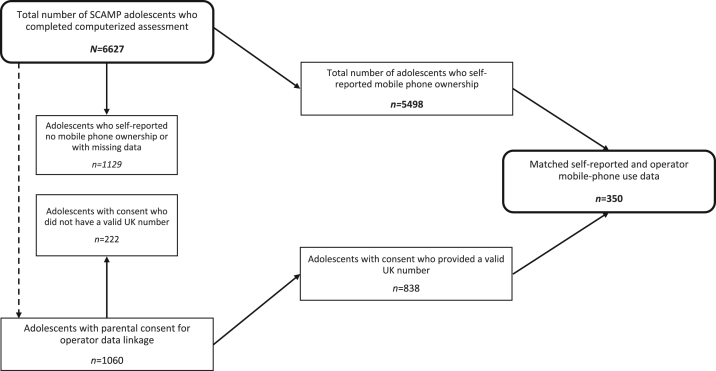
SCAMP study flow chart of sample selection for the validation study. (----Parental consent for data linkage is requested for all SCAMP children irrespective of mobile phone ownership).

Of 1060 parents of SCAMP adolescents who gave consent to access mobile operator traffic records, 838 provided a valid UK number for the adolescent. The remaining 222 did not use mobile phones, provided no phone number or provided an invalid UK phone number. Network operators successfully matched data for 355 adolescents (42.4%). One of the matched data were excluded from the analysis because it was a duplicate. Further, we excluded four matched numbers because self-reported mobile data were missing; therefore, data analysis was based on n = 350 as shown in Fig. 1.

Mobile network operators provided traffic data for voice calls (call date, duration, start/end times of the data period), text messages (text date, incoming/outgoing, start/end times of the data period), and mobile data use (data use in kb, date, start/end time of the data period). The three time periods for which data were provided were February-July 2016 (operator #1), June-August 2015 (operator #2), and July-August 2015 (operator #3). These windows all correspond to a 3 month period (June-July 2015, February-April 2016, and May-July 2016) and during which baseline data collection was ongoing. However, as mobile network operators in the UK retain data for three to six months only, in some cases, the mobile operator traffic data period did not overlap with the time period during which the adolescents in the sub-study self-reported their use. Overlap (date of self-reported mobile phone usage within the period for which traffic data were received) between the self-reported usage period and the mobile operator traffic data was achieved in 12.1% of adolescents.

Mobile operator traffic data were cleaned and summarised into daily rates for number of phone calls made on weekdays/weekends, overall call time or duration, text messages sent and mobile data downloaded. These data were then allocated into the same categories and time periods as the self-reported mobile phone use data. Two of the operators did not specify whether text messages were incoming or outgoing, so to match with self-reported values of outgoing messages, the operator text message frequencies were halved^3^ (Gold et al., 2015). The mobile operator traffic data from August 2015 showed significantly lower mobile phone usage in terms of the total number of phone calls made (*P*-value< 0.001) and cumulative duration of call time (*P*-value< 0.001) in comparison to other months; thus, August data were excluded from the analysis to avoid potential bias from differential phone use during, and possibly due to, the summer vacation break in the UK.

### 2.4 Statistical analysis

Socio-demographic characteristics and self-reported mobile phone use were compared between the full subset of the SCAMP cohort with parental consent and who used mobile phones and the operator subset. Pearson chi-square tests were used to compare the characteristics between the two groups except where the cell count was less than 5, in which case Fisher's exact test was used. Wilcoxon rank sum test was used to compare the distribution of age between the two groups.

The percentage of perfect agreement, underestimation and overestimation of the daily frequency of phone calls made, daily duration of speaking on the phone, and number of text messages sent on weekdays and weekends was evaluated by cross tabulation. Cohen's weighted kappa (ĸ) statistics, with even weighting between response categories, was used to evaluate the reliability of questionnaire responses on mobile phone use. The ĸ- values ≤ 0, 0.01–0.20, 0.21–0.40, 0.41–0.60, 0.61–0.80, and 0.81–1.0 indicate poor, slight/low, fair, moderate, substantial, and near-perfect agreement, respectively (Landis and Koch, 1977). No mobile phone use as indicated in either network or self-reported data was set to a value of “0” (Aydin et al., 2011b). Call frequency was defined as low if < 1/day and high if ≥ 11/day. Call duration was defined as low if ≤ 5 min/day and high if > 30 min/day. Number of text messages sent was defined as low if ≤ 5/day and high if ≥ 11/day. The sensitivity and specificity of the questionnaire data and 95% confidence intervals (CI) to accurately report low or high mobile phone use (using mobile operator traffic data as the gold standard) were measured separately for weekdays and weekends.

We further assessed the extent of agreement between the self-reported and mobile operator traffic data stratified by socio-demographic factors. Statistical significance was defined as *P*-value less than 0.05 from two-tailed tests. All statistical analyses were performed on the SCAMP dataset frozen at August 2016 using STATA version 13 (StataCorp, College Station, TX).

## 3 Results

The median age (interquartile range) of the operator subset was 12 (11.7—12.3) years and most of the children (71.2%) in the operator subset were of a high socio-economic status. Overall, socio-demographic characteristics were similar between the operator subset and the rest of the consented cohort who used mobile phones, apart from a slight difference in the ethnicity mix of borderline significance. Similarly, there were no differences between the two groups in terms of self-reported mobile phone use (Table 1).

**Table 1:** Comparison of adolescent characteristics and self-reported mobile phone use.

**Characteristics**		**Operator subset (n = 350)**	**Rest of consented group**^b^	***P*****-value**
			**(n = 488)**	
		n (%)	n (%)	
**Age (years)**^a^		12 (11.7–12.3)	12 (11.8–12.3)	0.059
**Sex**	Male	145 (41.4)	225 (46.1)	0.179
	Female	205 (58.6)	263 (53.9)	
**SES**	High	249 (71.2)	353 (72.3)	0.866
	Medium	54 (15.4)	69 (14.1)	
	Low	26 (7.4)	35 (7.2)	
	Missing	21 (6.0)	31 (6.4)	
**Ethnicity**	White	222 (63.4)	276 (56.6)	0.050
	Black	19 (5.4)	33 (6.8)	
	Asian	31 (8.9)	66 (13.5)	
	Mixed	41 (11.7)	44 (9.0)	
	Other	20 (5.7)	41 (8.4)	
	Missing	17 (4.9)	28 (5.7)	
**Weekday use**				
**Call Frequency**^c^	< 1	140 (40.0)	205 (42.0)	0.343
	~1	100 (28.6)	125 (25.6)	
	2–5 times	91 (26.0)	96 (19.7)	
	6–10 times	13 (3.7)	25 (5.1)	
	11–20 times	5 (1.4)	6 (1.2)	
	> 21 times	1 (0.3)	4 (0.8)	
	Missing	0 (0)	27 (5.6)	
**Call duration**^d^	0 min	32 (9.1)	50 (10.3)	0.774
	1–5 min	191 (54.6)	255 (52.3)	
	6–15 min	81 (23.1)	88 (18.0)	
	16–30 min	25 (7.1)	36 (7.4)	
	31–59 min	7 (2.0)	13 (2.7)	
	1–2 h	9 (2.6)	10 (2.1)	
	> 3 h	5 (1.4)	9 (1.8)	
	Missing	0 (0)	27 (5.5)	
**Text messages**^e^	< 1	38 (10.9)	82 (16.8)	0.097
	1–5	147 (42.0)	181 (37.1)	
	6–10	82 (23.4)	88 (18.0)	
	11–40	55 (15.7)	69 (14.1)	
	41–70	13 (3.7)	23 (4.7)	
	71–100	6 (1.7)	10 (2.1)	
	> 100	9 (2.6)	7 (1.4)	
	Missing	0 (0)	28 (5.7)	
**Weekend use**				
**Call Frequency**^c^	< 1	159 (45.4)	215 (44.1)	0.400
	~1	74 (21.1)	108 (22.1)	
	2–5 times	78 (22.3)	76 (15.6)	
	6–10 times	22 (6.3)	32 (6.6)	
	11–20 times	11 (3.1)	20 (4.1)	
	> 21 times	6 (1.7)	10 (2.1)	
	Missing	0 (0)	27 (5.5)	
**Call duration**^d^	0 min	48 (13.7)	77 (15.8)	0.716
	1–5 min	137 (39.1)	185 (37.9)	
	6–15 min	87 (24.9)	97 (19.9)	
	16–30 min	32 (9.1)	45 (9.2)	
	31–59 min	24 (6.9)	24 (4.9)	
	1–2 h	11 (3.1)	16 (3.3)	
	> 3 h	11 (3.1)	17 (3.5)	
	Missing	0 (0)	27 (5.5)	
**Text messages**^e^	< 1	57 (16.3)	94 (19.3)	0.456
	1–5	101 (28.9)	148 (30.3)	
	6–10	65 (18.6)	76 (15.6)	
	11–40	74 (21.1)	76 (15.6)	
	41–70	24 (6.9)	33 (6.8)	
	71–100	14 (4.0)	15 (3.1)	
	> 100	15 (4.3)	18 (3.7)	
	Missing	0 (0)	28 (5.7)	

Missing categories were not used in the comparison of proportions between the two groups.

a Median (Inter quartile range).

b The rest of the SCAMP cohort who had parental consent and provided a valid UK number.

c Call frequency refers to only the self-reported number of calls made per day.

d Call duration refers to the self-reported duration of calls made per day.

e Text messages refer to only the self-reported number of text messages sent (outgoing) per day.

Table 2 shows the comparison of operator to self-reported mobile phone use among adolescents on weekdays (A) and weekends (B). Perfect agreement between self-reported and operator–derived mobile phone use was highest for the duration spent talking on mobile phones per day on weekdays (38.9%) and weekends (29.4%) compared to frequency of calls and number of text messages sent. In general, adolescents overestimated their call duration and number of text messages sent per day both on weekdays and weekends (Table 2). In contrast, adolescents underestimated the frequency of calls they made per day on weekdays and weekends. As shown in Table 2, adolescents generally overestimated their mobile phone use during weekends in comparison to their mobile phone use during weekdays. There was slight agreement between self-reported and mobile operator traffic data for call frequency and call duration during weekdays (κ = 0.12, 95% CI 0.06–0.18 and κ = 0.08, 95% CI: 0.03–0.15, respectively) but poor agreement for the number of text messages sent (Table 3). A similar pattern of agreement between self-reported and mobile operator traffic data was observed for mobile phone use during weekends.

**Table 2:** Comparison of self-reported mobile phone use to operator traffic data.

Sections highlighted indicate higher proportion of underestimation or overestimation.

**Table 3:** Agreement, sensitivity and specificity of self-reported mobile phone-use in comparison with objective operator traffic data.

N = 350	**Weighted Kappa**	**Sensitivity (95% CI)**		**Specificity (95% CI)**	
	**(95% CI)**	**Low use (%)**	**High use (%)**	**Low use (%)**	**High use (%)**
**During weekdays**					
Call Frequency^a^	0.12 (0.06–0.18)	77.1 (69.3–83.7)	3.2 (0.1–16.7)	37.4 (30.8–44.4)	98.4 (96.4–99.5)
Call Duration^b^	0.08 (0.03–0.15)	66.8 (61.0–72.2)	12.5 (0.3–52.7)	49.3 (36.8–61.8)	94.2 (91.1–96.4)
Text messages^c^	0.01 (−0.04–0.06)	53.1 (47.2–59.0)	10.7 (2.27–28.2)	48.4 (35.5–61.4)	75.2 (70.1–79.8)
**During weekends**					
Call Frequency^a^	0.13 (0.06–0.20)	72.0 (65.0–78.4)	13 (2.78–33.6)	39.6 (32.1–47.6)	95.7 (92.9–97.6)
Call Duration^b^	0.10 (0.05–0.15)	56.1 (50.2–61.8)	0.0 (0.0–84.2)	64.8 (50.6–77.3)	86.8 (82.8–90.2)
Text messages^c^	0.03 (−0.02–0.07)	45.5 (39.9–51.2)	40. 9 (20.7–63.6)	57.9 (40.8–73.7)	64.0 (58.6–69.2)

a Call frequency refers to only the average number of calls made per day.

b Call duration refers to the average duration of calls per day.

c Text messages refer to only the average number of text messages sent (outgoing) per day.

Analysis of agreement between the self-reported and mobile operator traffic data usage on weekdays (Table 4) and weekends (Supplementary Table 1), stratified by socio-demographic factors, showed little difference overall between the sexes and socio-economic groups. There was, however, a slight tendency for agreement to be poorer amongst females. For ethnicity, black adolescents had moderate reliability for weekday call frequency and call duration (κ = 0.43, 95% CI 0.21–0.68 and κ = 0.28, 95% CI: 0.07–0.55, respectively) which was considerably higher reliability than for all other ethnic groups. Moreover, those of mixed ethnicity had considerably lower reliability for text messaging on weekdays and weekends (κ =−0.15, 95% CI −0.28 to −0.01 and κ = −0.07, 95% CI −0.22 to 0.09 respectively) than all other ethnic groups (Table 4 and Supplementary Table 1).

**Table 4:** Agreement of self-reported mobile phone use on weekdays in comparison with objective operator traffic data by socio-demographic characteristics.

	**Category**	**Number**	**Weighted Kappa (95% CI)**
**Call Frequency**^a^			
Sex	Male	145	0.16 (0.06 −0.26)
	Female	205	0.08 (0.01–0.16)
SES^b^	High	249	0.11 (0.05–0.19)
	Medium	54	0.07 (−0.07 to 0.21)
	Low	26	0.15 (−0.1 to 0.42)
Ethnicity	White	222	0.12 (0.04–0.18)
	Black	19	0.43 (0.21–0.68)
	Asian	31	0.15 (−0.06 to 0.45)
	Mixed	41	0.08 (−0.06 to 0.23)
	Other	20	0.18 (−0.04 to 0.43)
**Call duration**^c^			
Sex	Male	145	0.10 (−0.02 to 0.24)
	Female	205	0.06 (−0.01 to 0.14)
SES^b^	High	249	0.04 (−0.02 to 0.12)
	Medium	54	0.12 (−0.07–0.33)
	Low	26	0.14 (0.00–0.33)
Ethnicity	White	222	0.05 (−0.02 to 0.13)
	Black	19	0.28 (0.07–0.55)
	Asian	31	0.13 (−0.04 to 0.32)
	Mixed	41	−0.01 (−0.16 to 0.17)
	Other	20	0.07 (−0.11 to 0.42)
**Text messages**^d^			
Sex	Male	145	0.01 (−0.06 to 0.10)
	Female	205	−0.02 (−0.08 to 0.06)
SES^b^	High	249	−0.01 (−0.06 to 0.05)
	Medium	54	0.14 (−0.02 to 0.32)
	Low	26	−0.02 (−0.07 to 0.00)
Ethnicity	White	222	0.03 (−0.03 to 0.09)
	Black	19	0.00 (−0.10 to 0.13)
	Asian	31	0.08 (−0.02 to 0.24)
	Mixed	41	−0.15 (−0.28 to −0.01)
	Other	20	0.00 (−0.20–0.19)

a Call frequency refers to only the average number of calls made per day.

b SES- Socioeconomic status.

c Call duration refers to the average duration of calls per day.

d Text messages refer to only the average number of text messages sent (outgoing) per day.

In general, the questionnaire on mobile phone use was relatively more sensitive for low users (< 1 call/day, ≤ 5 min of call/day or ≤ 5 text messages sent/day) and more specific for high users (≥ 11 calls/day, > 30 min of call/day or ≥ 11 text messages sent /day). Specifically, of the three modes of mobile phone use measured in the questionnaire, call frequency was the most sensitive for low mobile phone users on weekdays and weekends (77.1, 95% CI: 69.3–83.7 and 72.0, 95% CI: 65.0–78.4, respectively). Specificity of questionnaire was moderate to high for high users with the highest for call frequency during weekdays (98.4, 95% CI: 96.4–99.5). Sensitivity for low and specificity for high users were lowest for the number of text messages sent on weekdays (53.1, 95% CI: 47.2–59.0 and 75.2, 95% CI: 70.1–79.8, respectively).

## 4 Discussion

This study is one of the first to assess the validity of self-reported mobile phone use in adolescents in the smart-phone era while taking into account weekday and weekend use separately. Overall, we found underestimation of call frequency, and overestimation of call duration and text messaging. Nevertheless, sensitivity and specificity for call frequency were moderate to high indicating that self-reported data allow clear differentiation between low and high usage.

Our findings coincided with the lower range of agreement in other validation studies (Inyang et al., 2009, Samkange-Zeeb et al., 2004). Approximately half of the number of calls made on weekdays or weekends were underreported, which supports earlier findings of call underreporting of call frequency (Timotijevic et al., 2009). In adolescent populations, studies assessing the validity of self-reported mobile phone use have compared with the software-modified phones (SMPs) as the “gold standard” (Inyang et al., 2009, Kiyohara et al., 2015) and the results are consistent with our findings. The time spent speaking on the phone was overestimated by about half of the individuals, a trend that is consistent with most research in both adolescents and adults (Goedhart et al., 2015, Parslow et al., 2003). More than half of adolescents overestimated the number of text messages sent, with a stronger overestimation trend on weekends compared to weekdays. This corroborates the findings of other studies also using billing records (Redmayne et al., 2012, Gold et al., 2015) to ascertain objective phone usage. A possible explanation for the more common propensity to underestimate the number of calls made, as opposed to the duration of calls, is that brief calls may not be as memorable and may lead to omission in recall estimates (Vrijheid et al., 2009).

This study also assessed the sensitivity and specificity of self-reported usage compared to the operator traffic data for both low and high users. For adolescents, the questionnaire showed a higher sensitivity for low mobile phone users than high users but higher specificity for high users. The findings suggest that the questionnaire is unlikely to misclassify true low mobile phone users but that it may miss a small number of true high mobile phone users. The observed sensitivity of the questionnaire for self-reported call frequency for low users in this study was higher than that reported for the only similar study among Year 7 adolescents in Australia thus 77% vs 57% (Inyang et al., 2009). Our contrasting sensitivity and specificity findings for low and high users might in part be explained by individuals using logarithmic scales mentally for higher numbers, in essence providing inaccuracy proportional to the magnitude of quantities estimated (Redmayne et al., 2012).

For all three mobile phone usage types on weekdays, females were found to have lower recall accuracy. Research has shown females to underestimate their number of calls (Abeele et al., 2013) and text messages (Boase and Ling, 2013) and overestimate duration of calls (Aydin et al., 2011b). Black adolescents in our cohort reported their call frequency more accurately compared to other ethnicities. These findings vary with the only other validation study identified that included ethnicity, which did not find any association of reporting accuracy with ethnicity (Inyang et al., 2009). However, sample sizes were small in the aforementioned study, leading to wide confidence intervals. These trends might represent a tendency for certain adolescents to exaggerate their mobile phones use in order to emphasize their sociability and/or ‘streetwise’ credibility (Leung and Wei, 2000). Alternatively, although very unlikely, it might also reflect some form of participation bias relative to the main SCAMP cohort, whereby high/low users in these subgroups were more likely to permit access to their children's mobile phone records.

In terms of self-reporting, with the myriad functions now provided by smartphones, there might be some confusion or inconsistency as to how individuals perceive and report different usage. For instance, nowadays, quantitative reports of sending instant messages or using social media network sites may be mixed up by participants with reports of sending SMS text messages (Berolo et al., 2015). Moreover, recent research indicates that heavier voice and SMS users are more likely to replace mobile network voice calls and SMS messages with calls and messages over mobile internet (data) usage, possibly due to higher cost savings and unlimited ‘data packages’ on offer from mobile network operators (Gerpott and Meinert, 2016). Hence, it would be more difficult to tease out accurate call frequency/duration and messaging agreement between self-reported and mobile operator traffic data for such individuals. In addition, a further complicating factor is the use of voice call applications and social media messaging over Wi-Fi networks. Such usage over Wi-Fi would not be evident in either the mobile network call time or data use information. In light of the above, providing categories of usage instead of open questions to collect continuous data as well as enquiring about other uses of a smartphone, as was done in this study, can help to improve self-reported accuracy and remove extreme values (Boase and Ling, 2013). For the SCAMP cohort, detailed self-reported data on other uses of mobile phones or usage over Wi-Fi networks, both of which are not captured in the mobile operator traffic data, are available and will facilitate accurate estimation of an adolescent's total usage of, and RF-EMF exposures from, mobile phones.

### 4.1 Strengths and limitations

A key strength of this study is the larger sample size compared to many other validation studies among adolescents and young adults which involved less than 200 participants (Inyang et al., 2009, Kiyohara et al., 2015). The similarity of socio-demographic characteristics and self-reported mobile phone use amongst SCAMP adolescents for whom we could obtain mobile network traffic data and the rest of the consented subset of the cohort, confirmed the study sample's representativeness. A further strength of this study is that it is the first to assess the validity of mobile phone data collected separately for weekdays and weekends and our results clearly show differences in agreement between uses of mobile phones on weekdays versus weekends. A benefit of using operator records to validate adolescents’ mobile phone use is that there is no additional occupational phone use, which has been shown to inflate self-reported behaviours (Kobayashi and Boase, 2012). At least one month of mobile network traffic data were available for all adolescents, which is as long or longer than reference datasets seen in earlier research (Abeele et al., 2013, Boase and Ling, 2013, Gold et al., 2015).

A potential limitation of the study was the 42.4% matching rate of phone numbers for the consented group by mobile operators; a lower matching rate than for similar adult mobile phone user cohort studies in the UK (Toledano et al., 2015). This is most likely due to the following scenarios: (1) different names for adolescents and the parental account holder, (2) mismatched names on network operator accounts and those recorded in SCAMP school assessments, (3) frequent porting/churning of mobile phone network operators over time (4) incorrect number recorded or (5) mobile phone network operator did not participate in research. Furthermore, the questionnaires did not ask for time-specific mobile phone usage which could have introduced some exposure misclassification into the self-reported mobile phone use data. This type of non-differential misclassification is common in cohorts with questionnaire data that require recall of past activities. Another limitation was the heterogeneity of the mobile operator traffic data received, which included varied periods of time, as well as misaligned calendar periods between self-reported and operator records. The percentage overlap between the periods when self-reported mobile phone use data was collected versus the period for which mobile operator traffic data was obtained was relatively small. This may explain some of the disagreement between the self-reported and operator recorded data. However, the same happens in an epidemiological study addressing long term health risk. Reporting may be affected by short term fluctuations in mobile phone use but is still then considered as a long term exposure surrogate. Thus, our findings are relevant for interpreting of corresponding research. (Aydin et al., 2011b, Timotijevic et al., 2009). Finally, the method of sampling for this validation study may possibly lead to non-participation bias. This is because parents of high socio-economic status and those who are Caucasian are more likely to provide consent for data linkage. However, in comparison with the rest of the children who used mobile phones and whose parents had provided consent, children in the operator subset had similar socio-demographic characteristics and self-reported mobile phone use.

Calls and therefore, overall call duration on handsets via mobile networks, are the most important contributor to an individual's RF-EMF exposure from their mobile phone. Therefore mobile network traffic data should still be best to validate such exposures (Lauer et al., 2013). However, as previously noted, smartphones are used in complex ways today, and it has become increasingly difficult for participants to accurately recall their different types of usage and differentiate which usage was over mobile network voice calls, SMS messages, or data and/or over Wi-Fi. In the SCAMP cohort, 95.6% of adolescents reported using the internet actively on their phones via Wi-Fi connection and not via the mobile network connection. Therefore, we would not expect mobile operator data to accurately reflect total usage or to show high agreement with self-reported usage in adolescents. Smartphone applications are now available to monitor all call and message use, including differentiation of those undertaken over mobile or Wi-Fi networks, and have started to be used in validation studies (Goedhart et al., 2015). The SCAMP study is now using one such application to obtain more detailed mobile phone use from another subset of the cohort; these results will be integrated with self-reported and operator traffic data to further elucidate patterns of mobile phone use amongst adolescents.

## 5 Conclusions

Our findings suggest differential agreement between adolescents’ self-reported mobile phone use and mobile operator traffic data during weekdays and weekends, by type of usage, and ethnicity. Adolescents underestimate the frequency of calls made but overestimate the duration of calls and number of text messages sent. Self-reported mobile phone use during weekends is generally overestimated in comparison with weekday mobile phone use. The moderate to high sensitivity and specificity for call frequency indicates that questionnaire for self-reported mobile phone use adequately distinguishes between low and high usage.

The greater use of mobile smartphones over Wi-Fi networks by adolescents, as opposed to mobile phone networks, means operator data are not the gold standard for exposure assessment in this age group. This has important implications for epidemiologic research on the health effects of mobile phone use in adolescents. To improve the accuracy of mobile phone usage estimation in epidemiological studies of adolescents in the current smartphone era, we recommend combining analysis of self-reported usage over Wi-Fi and mobile operator traffic data.

## Footnotes

1 1 “Higher managerial, administrative and professional occupations”; 2 “Intermediate occupations”; 3 “Small employers and own account workers”; 4 “Lower supervisory and technical occupations”; and 5 “Semi-routine routine occupations”.

2 1 “Higher managerial, administrative and professional occupations”; 2 “Intermediate occupations”; and 3 “Routine and manual occupations”.

3 46.0% of text messages were sent in the operator data that differentiated between messages sent or received.
