# Supplementary material for: Total recall in the SCAMP cohort: Validation of self-reported mobile phone use in the smartphone era
Source: Environ Res. 2018 Feb;161:1–8. doi: 10.1016/j.envres.2017.10.034 (PMC5773244; doi:10.1016/j.envres.2017.10.034)
Supplement: Supplementary file 1 — Supplementary material [file mmc1.docx]

Supplementary Table 1. Agreement of self-reported mobile phone use on weekends in comparison with objective operator traffic data by socio-demographic characteristics

|  | **Category** | **Number** | **Weighted Kappa (95% CI)** |
| --- | --- | --- | --- |
| **Call Frequency^a^** |  |  |  |
| Sex | Male | 145 | 0.16 (0.07-0.28) |
|  | Female | 205 | 0.11 (0.02-0.21) |
| SES^b^ | High | 249 | 0.12 (0.04-0.22) |
|  | Medium | 54 | 0.19 (0.03-0.38) |
|  | Low | 26 | 0.00 (-0.23-0.26) |
| Ethnicity | White | 222 | 0.11 (0.02-0.21) |
|  | Black | 19 | 0.37 (0.07-0.61) |
|  | Asian | 31 | 0.11 (-0.15-0.35) |
|  | Mixed | 41 | 0.13 (-0.07-0.35) |
|  | Other | 20 | 0.23 (-0.17-0.58) |
|  |  |  |  |
| **Call duration^c^** |  |  |  |
| Sex | Male | 145 | 0.07 (-0.00-0.14) |
|  | Female | 205 | 0.11 (0.05-0.18) |
| SES^b^ | High | 249 | 0.09 (0.04-0.15) |
|  | Medium | 54 | 0.14 (0.01-0.28) |
|  | Low | 26 | 0.02 (-0.00-0.09) |
| Ethnicity | White | 222 | 0.11 (0.06-0.17) |
|  | Black | 19 | 0.07 (-0.03-0.27) |
|  | Asian | 31 | 0.01 (-0.11-0.15) |
|  | Mixed | 41 | 0.08 (-0.04-0.23) |
|  | Other | 20 | 0.19 (-0.10-0.46) |
|  |  |  |  |
| **Text messages^d^** |  |  |  |
| Sex | Male | 145 | 0.02 (-0.03-0.07) |
|  | Female | 205 | 0.03 (-0.03-0.10) |
| SES^b^ | High | 249 | 0.00 (-0.05-0.05) |
|  | Medium | 54 | 0.08 (-0.01-0.22) |
|  | Low | 26 | 0.02 (-0.04-0.12) |
| Ethnicity | White | 222 | 0.04 (-0.02-0.09) |
|  | Black | 19 | 0.01 (-0.08-0.09) |
|  | Asian | 31 | 0.03 (-0.06-0.14) |
|  | Mixed | 41 | -0.07 (-0.22-0.09) |
|  | Other | 20 | 0.06 (-0.10-0.28) |

^a^Call frequency refers to only the average number of calls made per day;  ^b^SES- Socioeconomic status; ^c^Call duration refers to the average duration per day; ^d^Text messages refer to only the average number of text messages sent (outgoing) per day
